# Serial changes in renal allograft resistive index

**DOI:** 10.1590/2175-8239-JBN-2020-E003

**Published:** 2020-11-30

**Authors:** Frank K Chen, Rupan Sanyal

**Affiliations:** 1 Mayo Clinic, Department of Radiology, Jacksonville, FL, USA.

Doppler ultrasound is widely used to identify complications following renal transplantation. Evaluation of renal arterial Doppler waveform involves assessment of the peak systolic velocity (PSV), acceleration time or rapidity of the upstroke, and the renal resistive index (RI). The RI measurement is obtained by dividing the difference between PSV and end diastolic velocity by the PSV. The resulting ratio eliminates the inconsistencies commonly encountered in measuring velocities due to inaccurate angle correction and can be considered a measure of vascular impedance. An RI > 0.8 is considered elevated.

In this issue, Moura-Neto et al^1^ evaluated the role of serial RI in the immediate postoperative period to assess delayed graft function due to ischemia-reperfusion injury in 113 renal transplant patients. The study excluded patients with histology-proven acute rejection, hydronephrosis of grade 2 or higher, and peritransplant collections causing marked compression, as these conditions may also cause elevated RIs. The authors measured the intrarenal RI in the first postoperative week and then again within thirty days post-transplant. They found that a subset of patients with deceased-donor renal transplantation had serial increase in RI in the first few weeks. Such increase was not seen in patients with living-donor transplants. This serial increase in RI was associated with delayed graft function, more severe ischemia-reperfusion injury, and the need for dialysis in the immediate post-operative period. This is consistent with studies that have shown RI elevation on immediate postoperative Doppler ultrasound as a predictor of acute tubular necrosis (ATN) and delayed graft function^2^^,^ 3. While most studies evaluate RI on a single early postoperative Doppler ultrasound, Moura-Neto et al^1^ incorporated two serial measurements of RI over four weeks and evaluated interval change. This approach of analyzing change over serial studies has been used by other authors and may be more reflective of the allograft characteristics than a single measurement, particularly in the early postoperative period.

Multiple studies have shown that a normal RI in the immediate post-operative period is a good predictor for immediate graft function. Moura-Neto et al^1^ add to this literature by reporting that decreasing RI in the first few post-operative weeks is associated with better serum creatinine and lower likelihood of requiring dialysis. 

RI elevation (RI > 0.8) can be seen in various causes of graft dysfunction including, but not limited to, ATN due to ischemia reperfusion injury, rejection, obstruction, and compression^4^. In the first few weeks after transplantation, ATN is the most common cause of elevated RI. Elevated RI with reversed diastolic flow is a sign of extremely high vascular resistance and correlated with high risk of graft loss. Reversed diastolic flow in the immediate post-operative period can be caused by ATN and acute rejection but also by potential surgically reversible conditions like renal vein thrombosis, compression by hematomas, or vascular kinks^5^.

The role of RI in identifying graft dysfunction has been studied with conflicting results. Most studies suggest that while evaluation of RI has not been able to identify specific causes of early graft dysfunction, elevated RI is significantly correlated with an increased incidence of delayed graft function. Even then, evaluation of RI falls short of effectively predicting the occurrence of graft dysfunction with only a moderate degree of estimated diagnostic accuracy^6^.

Elevated RI not only results from intrarenal factors but may also be secondary to systemic factors, such as heart rate, patient age, hypotension, and aortic stiffness. In a study by Naesens et al^7^, RI was evaluated at predetermined time-points of 3, 12, and 24 months after transplantation during protocol biopsies and the authors found that RIs reflected the recipient characteristics like older age and not renal allograft histological features. In the same study, RI measured at time of biopsies for graft dysfunction were significantly higher than RI measured at time of protocol biopsies, corroborating the association of RI elevation and graft dysfunction noted in other studies. 

In this study, the authors evaluated the changes of RI over two serial Doppler ultrasound examinations obtained within the first thirty days post-transplant. In current practice, the majority of post-renal transplant patients will only have one post-transplant ultrasound study performed in the immediate post-transplant period. It is unclear if it is feasible or practical to adopt the use of serial Doppler ultrasound examinations in all post-transplant patients. If not suitable for all renal transplant patients, it is necessary to determine which subset of patients would benefit from serial examinations and how often and when the serial studies should be performed. Further studies are also required to evaluate the efficacy of serial ultrasounds in predicting delayed graft function compared to clinical and biochemical markers.

To conclude, the study by Moura-Neto et al^1^ showed an association between increases in RI on serial Doppler studies in the first few post-operative weeks and delayed graft function due to ischemia-reperfusion injury. Serial evaluations could better identify patients at risk for delayed graft function than RIs measured on a single early post-operative Doppler examination and allow for prompt prevention and intervention.
